# Excessive iodine status among school‐aged children in the State of Qatar: Results of the National Iodine Deficiency Disorder Survey

**DOI:** 10.1002/puh2.60

**Published:** 2023-02-20

**Authors:** Mohamed Hamad J. T. Al‐Thani, Salah Abdulla Sh. A. Alyafei, Kholoud Ateeq K. M. Al‐Motawaa, Shamseldin Ali Khalifa, Syed Hassan Bin Usman Shah, Benjamin Vinodson, Sureshbabu Kokku, Amit Mishra

**Affiliations:** ^1^ Public Health Department Ministry of Public Health Doha Qatar; ^2^ The Kirby Institute University of New South Wales Kensington Australia

**Keywords:** iodine deficiency, iodine excess, salt iodization, State of Qatar, urinary iodine

## Abstract

**Background:**

Iodine deficiency poses a significant public health challenge worldwide, particularly in preschool children and pregnant women. Assessing the iodine intake at a population level is essential, as both deficient and excessive iodine status can have adverse health consequences. The main objective of this survey is to understand the iodine deficiency status in schoolchildren aged 6–12 years in the State of Qatar.

**Methods:**

A cross‐sectional survey was conducted from March to June 2014 among school‐aged children using a two‐stage cluster sampling technique. Anthropometric, biochemical, clinical, and dietary parameters for seafood were collected. Spot urine samples were collected from 967 participants, and a repeat random subsample urine was collected from 288 participants. Overall median urinary iodine concentration (UIC) was calculated. Iodine content in household salt samples was estimated by qualitative and quantitative methods. Mann–Whitney *U* test was used for comparison.

**Results:**

The median UIC was 333.2 μg/L (IQR = 228.6). UIC prevalence rates between 300–999 and >1000 μg/L were 56.7% and 2.8%, respectively. The goiter prevalence was 0.4%. A significant difference was noted in overall median UIC levels between boys and girls (*p* = 0.003). Adequate iodized salt consumption was reported by 74.9% of households, and weekly seafood consumption was reported by one third of the respondents.

**Conclusion:**

The results indicate an excess intake of iodine among the studied population in the State of Qatar, and national efforts are needed to bring iodine intake and concentration levels within the cutoff value for the concerned survey population's age group. In addition, a surveillance system needs to be set up for continuous monitoring of iodine content and salt intake at the population level in the State of Qatar.

## INTRODUCTION

Iodine is an essential trace element that has an important role in synthesizing the thyroid hormone in the human body [1]. Primary food sources of iodine include seaweed, seafood, dairy products, especially milk (if iodine is used in animal feed), eggs, and grain products. The Food and Nutrition Board at the Institute of Medicine of the National Academies recommends that children and adolescents between the ages of 1–8, 9–13, and 14–18 years require daily amounts of iodine 90, 120, and 150 μg, respectively [2].

Iodine is essential for normal body growth and fetal brain development; however, its deficiency or excess both have adverse health effects and may result in thyroid dysfunction either with or without goiter [3]. Iodine deficiency can have many adverse health effects and may lead to impaired thyroid hormone synthesis, resulting in functional and developmental abnormalities. These abnormalities will eventually progress into iodine deficiency disorders (IDD), which is recognized as a serious public health concern by the World Health Organization (WHO) [4, 5]. The consequences of Iodine deficiency disproportionately affect pregnant women, infants, and young children [6]. Evidence suggests that Iodine deficiency contributes to retarded psychomotor development and cretinism, low intelligence quotient, stillbirth and miscarriage, hypothyroidism, and even infant mortality [7, 8, 9]. Fortunately, iodine deficiency is among the least expensive nutrient deficiencies to prevent, owing to salt iodization [10]. On the other hand, excess iodine consumption can alter thyroid function in some people, particularly individuals with antithyroid antibodies [8, 11].

Long‐term and sustainable iodine prophylaxis can help prevent the adverse effects of iodine deficiency [12]. IDD's elimination goal was established during the United Nations World Summit for Children in 1990 [13]. Iodine can usually be obtained by consuming iodine‐rich food (like seafood and dairy products), supplementation, or food fortification, for example, iodized salt [14]. Salt, a low‐cost and widely available mineral, is the most used medium for iodization. Significant progress has been observed by WHO, the United Nations Children's Fund (UNICEF), and the International Council for the Control of Iodine Deficiency Disorders (ICCIDD), largely by adopting the universal salt iodization strategy [7, 13]. UNICEF estimates that about 90% of the world population is consuming iodized salt, with a range of disparities [15]. In many countries, Iodine intakes are sufficient among school‐aged children; however, iodine intakes among pregnant and reproductive‐age women are insufficient owing to their additional requirements [16].

The prevalence of IDDs in most of the WHO Eastern Mediterranean countries is currently under control, and literature indicates that a number of countries in this region have reached sufficient dietary iodine status in their population [7].

Measurement of population iodine status has been an important strategy adopted by WHO to monitor and control IDDs and address the emerging issue of higher iodine consumption. Several methods are used to monitor population iodine status [17]. Approximately 90% of the ingested iodine is excreted through the kidneys. The median spot urinary iodine concentration (UIC) is a sensitive indicator and an exceptional biomarker for recent dietary iodine consumption [5, 18]. Sampling UIC in school‐aged children can be used as a proxy indicator of population iodine status. Similarly, estimating iodized salt consumption by the household is also considered a proxy for population iodine status. Salt is considered adequately iodized if it contains 15–40 ppm of iodine [13]. The State of Qatar conducted its first‐ever study to determine the prevalence of IDD in 1996. Later in 2005, the World Health Assembly (WHA) requested national governments to update their iodine nutritional status every 3 years [19]. In‐line with this consensus, the State of Qatar conducted the second Qatar National Iodine Deficiency Disorder Survey (QNIDDS) in 2014 among 6–12‐year‐old school‐aged children to assess the iodine nutrition status. In addition, the survey aimed to identify the iodine content of household salt samples of respective school children and to assess the knowledge, attitudes, and practices of schoolchildren and their parents regarding iodine deficiency and its associated health consequences. This paper aims to outline the key findings of this survey.

## MATERIALS AND METHODS

### Study design, population, and sampling

QNIDDS was a cross‐sectional survey conducted between March and June 2014 among school‐aged children using a two‐stage cluster sampling technique. Schooling system in the State of Qatar is being regulated by the Ministry of Education and Higher Education (MEHE). For this study, MEHE provided a list of 98 schools to draw the sample. A two‐stage cluster sampling was done to identify the target population. In the first stage, 49 schools were selected, followed by a selection of classes/grades in the second stage. Out of 49 schools, 22 were all‐girls schools, and 27 were all‐boys schools. An entire class was chosen from each selected school in the second stage of sampling. On average, a class can have 25–30 students according to the recommendations of MEHE. The QNIDDS sample size was estimated based on an earlier IDD study conducted in 1996. In this survey, the incidence of less than 100 μg/L of median UIC was around 30%. Using these estimates and keeping the significance level at 95% and the desired precision at 0.05, the calculated sample size was 318. However, the sample size was inflated to 1193 students based on the 2007–08 WHO/UNICEF/ICCIDD guidelines [19], the design effect recommendation of three for this study and a predicted response rate of 80%.

### Study variables and data collection

To collect data, six data collection teams were formed, each comprising three health‐care professionals (a doctor, nurse, and assistant) alongside 30 field workers from the Ministry of Public Health (MoPH) and several volunteers. All data collectors received intensive 3‐day training before fieldwork. The study tool was developed after carefully reviewing the literature, which included three sections [13, 20, 21]. A set of questions regarding the sociodemographic profile were included in the first section. The second section had questions about using iodized salt and about goiter, whereas the third section determined parents’ awareness and attitudes toward using iodized salt. Information regarding the frequency of seafood consumption was also gathered.

Prior to the school visits, respective principals and nurses were communicated about the study purpose and methods, assuring them of strict confidentiality. The school administration and the survey team decided on data collection dates. Each school assigned a coordinator for team facilitation. Every selected student was provided with an envelope that included the survey protocol explaining its methodology, the questionnaire and informed consent form, and a plastic resealable bag for the collection of salt samples used in their household.

Anthropometric measurements were taken during the second visit. The height scale (seca 213) was used to measure the height without shoes and rounded to the nearest millimeter. While measuring weight, an 813 seca digital weight floor scale was used and recorded to the nearest 100 g after placing it on a firm and flat surface. The filled questionnaires, signed consent forms, and salt samples were collected during the second prearranged school visit. During this visit, the student's physical examination for the presence of both visible and palpable goiter was conducted by physicians according to the standard protocols. Parents were informed and advised to get the further medical investigation in case of pathologically enlarged thyroid gland detection on clinical examination. Moreover, during this visit, students also received proper instructions for providing spot urine samples, and the samples were collected.

The second spot urine sample was collected from randomly selected 288 students from the same cohort during the third visit. Once both urine samples were collected and coded, they were transferred at a recommended temperature to MoPH premises. The samples were transported to a WHO‐accredited laboratory in Tanzania for analysis.

The iodine content of salt was determined quantitatively by the titration method and using spectrophotometry, and qualitatively through the use of the rapid test kits (RTK) by placing two‐to‐three drops on each salt sample. Samples with no change in color were recorded as ‘0,’ indicating no iodine in the salt: However, salt samples that turned blue were recorded as ‘1,’ indicating the presence of iodine. The results were then recorded in the data collection form.

### Data analysis

The baseline characteristics and qualitative variable data of the students were reported using frequency distributions and descriptive statistics. Moreover, the Mann–Whitney *U* test was used to compare two groups that were not normally distributed. Median UIC levels were calculated using the recommendations based on WHO/UNICEF/ICCIDD guidelines [20]. According to the guideline, UIC < 20 μg/L is considered severe iodine deficiency, 20–49 μg/L moderate, and 50–99 μg/L mild iodine deficiency. A level of 100–199 μg/L is considered adequate; however, levels between 200 and 299 μg/L are considered a slight risk. Any value above 300 μg/L is considered a high risk, which may lead to adverse health consequences. All analyses were done using IBM SPSS Statistics for Windows, version 23.0 (IBM Corp., Armonk, NY, USA).

### Ethical considerations

Informed consent was taken from the parents/guardians as the target population included 6–12‐year‐old primary school‐going children (grades 1–6). Additionally, permission was taken from each school administration to undertake the survey. Ethical approval for this study was granted by the Research Ethics Board of the MoPH and the MEHE.

## RESULTS

### Study participants

Initially, a total of 1213 primary school students were selected. However, the sample was later reduced to 1195 as 18 students were transferred to other schools. A total of 1020 students out of 1195 agreed to participate in the survey, giving a response rate of 85.4%. Later, 8 students were excluded from the study due to age differences (more than 12 years). A four‐digit identification code was allotted to each student to keep the identity anonymous. In total, 967 students participated in the final analysis of this study, most of whom were boys (56.6% of the survey respondents). The mean age of the respondents was 8.8 ± 1.6 years (9.1 ± 1.7 and 8.7 ± 1.5 years for girls and boys, respectively). The distribution of students according to age and gender is given in Table 1.

**TABLE 1:** Age and gender distribution among study participants aged 6–12 years in Qatar in 2014

	Girls		Boys		Total	
Age (years)	No.	%	No.	%	No.	%
6	67	15.3	36	6.3	103	10.2
7	18	4.1	118	20.6	136	13.4
8	58	13.2	122	21.3	180	17.8
9	93	21.2	119	20.7	212	20.9
10	101	23.0	103	18.0	204	20.2
11	85	19.4	63	11.0	148	14.6
12	17	3.8	12	2.1	29	2.9
**Total**	439	100.0	573	100.0	1012	100.0
**Mean (age ± SD) and median (IQR)**	9.1 ± 1.7		8.7 ± 1.5		8.8 ± 1.6	

### Main results

A palpable/visible goiter (grades 1 and 2) was documented as positive upon a physical examination, which was found in 0.4% of the participants. More boys were affected by goiter compared to girls, 0.53% versus 0.23% (*p* = 0.637), respectively (Table 2).

**TABLE 2:** Total goiter rate among study participants aged 6–12 years

	Overall		Girls		Boys	
Category	No.	%	No.	%	No.	%
Positive	4	0.40	1	0.23	3	0.53
Negative	992	99.6	431	99.77	561	99.47
Chi square (0.637)						

Mann–Whitney *U* test results showed a significant difference between boys’ and girls’ median UIC levels {median UIC males = 351.1 (IQR = 245.7), median UIC females = 316.0 (IQR = 212.0); *p* = 0.003} (Table 3).

**TABLE 3:** Distribution of median urinary iodine concentration (UIC) among study participants by age and sex

	Overall UIC‐1st urine sample		Girls’ UIC‐1st urine sample		Boys’ UIC‐1st urine sample	
Age (years)	Median UIC (IQR) (μg/L)	No. of students	Median UIC (IQR) (μg/L)	No. of students	Median UIC (IQR) (μg/L)	No. of students
6	371.6 (289.8)	94	358.7 (291.1)	58	385.9 (308.4)	36
7	371.6 (289.8)	131	307.6 (263.3)	17	391.3 (265.9)	114
8	324.2 (217.0)	173	300.4 (179.3)	56	328.6 (221.2)	117
9	328.3 (214.0)	200	282.2 (208.5)	85	353.6 (238.8)	115
10	341.8 (220.1)	197	317.4 (176.0)	96	373.8 (255.3)	101
11	315.8 (231.0)	145	355.4 (271.4)	83	299.9 (181.8)	62
12	320.0 (262.6)	27	335.4 (245.8)	16	309.0 (256.4)	11
Overall	333.2 (228.6)	967	316.0 (212.0)	411	351.1 (245.7)	556

According to the WHO‐/UNICEF‐/ICCIDD‐recommended cutoffs, only 12.5% of students had a UIC falling within the optimal recommended range of 100–199 μg/L. The majority (81.1%) of the participants exceeded this suggested range, as shown in Table 4. The prevalence rates of UIC between 300–999 and >1000 μg/L were 56.7% and 2.8%, respectively.

**TABLE 4:** Distribution of urinary iodine concentration (UIC) among study participants according to the World Health Organization (WHO) cutoff points

		First urine sample	
UIC (μg/L)	Iodine status	No.	%
<20	Severe iodine deficiency	6	0.6
20–49	Moderate iodine deficiency	22	2.3
50–99	Mild iodine deficiency	34	3.5
100–199	Adequate iodine nutrition	121	12.5
200–299	Slight risk	207	21.4
300–399	Risk of adverse health consequences	224	23.2
400–499		149	15.4
>500		204	21.1
Total		967	100

Data on anthropometric measurements of 967 students was collected with a mean weight of 34.6 ± 12.7 kg and a median of 31.5 kg. Girls had a higher mean weight than boys (35.6 ± 12.9 and 33.9 ± 12.5 kg, respectively). The maximum overall recorded weight was 94.6 kg.

The median‐calculated UIC value for the first sample was 333.2 μg/L, and for the repeat sample, it was 360.4 μg/L, whereas the adjusted distribution median value was 341.6 μg/L for both samples. The urine subsamples were adjusted for intraindividual variation to obtain habitual iodine intake. These were then inferred using the estimated average requirement (EAR) cutoff point model. UIC cutoff values were calculated based on the median weight (31.5 kg) of the students. These values corresponded to an iodine EAR of 73 μg/day with a tolerable upper iodine intake level of 600 μg/day. According to the WHO guidelines [20], the median UIC falls in the ‘excessive intake’ group, whereas the adjusted distribution indicated habitual iodine consumption, which lies within the suggested range.

Almost half (49%) of RTK–analyzed salt samples (qualitative analysis) showed a positive iodization result, whereas results of the titration method (quantitative analysis) indicated a median iodine content of 25.30 ppm with a mean of 27.1 ± 13.6 ppm. As per the results of titration method, around 75% of households used iodized salt adequately, whereas 12.5% used iodized salt excessively (>40 ppm, according to the WHO guidelines). Based on median UIC (333.2 μg/L) and salt iodine content (25.3 ppm), the calculated salt intake (333.2/25.3 = 13.2 g/day) was around three times higher than the WHO recommendations of <2 g/day of sodium, which equates to 5 g/day of salt [15] (Table 5).

**TABLE 5:** Iodine content of the household salt samples collected from respondents

Iodine content in household salt	Number of samples	Percentage
No iodine	6	0.6
Inadequate iodine (<15 ppm)	119	12.0
Adequate iodine (15–40 ppm)	743	74.9
Excessive iodine (>40 ppm)	124	12.5
**Total**	**992**	**100.0**

This study revealed that around 55.1% of respondents were aware of the health benefits of iodine, with more than two thirds (*n* = 658, 68%) claiming that they have heard about goiter. Around 57.8% of the respondents read the packing labels, and 86.8% of participants claimed that the salt they use at home has an iodine label. Seafood that is considered a good source of iodine was consumed regularly (on a weekly basis) by one third of the respondent (31.3%) and once in 2 weeks by around 40% of participants. Around 30% of students reported eating fish‐/seafood‐containing meals a day before the urine sample was given, whereas *n* = 35 did not provide an answer to the question regarding seafood consumption a day before the urine sample collection.

## DISCUSSION

### Key findings

The QNIDDS (2014) is the most extensive study of its kind to calculate the prevalence of IDD among school‐going children involving more than 1000 students from 49 primary schools across the State of Qatar. If calculated using the appropriate sampling method, UIC is the most practical biochemical maker for measuring iodine status. Our results indicated that the median UIC of school‐aged children in the State of Qatar was 333.2 μg/L, which falls under the category of excessive iodine status based on the criteria established by WHO [13, 19]. Studies conducted by Choi et al. and Kang et al. in Korean school children showed a median UIC of 438.8 and 449 μg/L, respectively, and these values correspond to iodine excess [11, 22]. In some iodine excess areas, the median UIC of more than 1000 μg/L has been reported [23]. Similarly, a review article on Cambodian school‐aged children's iodine status showed median UIC levels between 200 and 299 μg/L, indicating more than adequate iodine status [24]. Another Chinese study reported iodine excess with a median UIC of 750.18 μg/L [3]. The present study showed that around 60% of our study participants had UIC values >300 μg/L, which is much better than 77% and 96% of Korean and Chinese students, respectively [22, 25]. Lee et al. reported that 66% of Korean school children have UIC of >300 μg/L, which is coherent with our study results [26].

Several reports state that females are more susceptible and are prominently diagnosed with thyroid disorders [8, 24, 25]. However, a significant difference in the median UIC levels of boys and girls was reported in our study, with boys having higher UIC levels. Similar findings were reported in an Ethiopian study [27]. However, these results contradict Kang et al. study, which found no difference between males’ and females’ UIC levels [11].

The calculated salt intake was around three times higher than the WHO recommendations of <2 g/day of sodium, which equates to 5 g/day of salt [13]. WHO recommends a salt reduction in all age groups, including in children. The maximum cutoff of 2 g/day of sodium intake should be adjusted and reduced even further based on the energy (kcal) requirements of the children between the ages of 2 and 15 years [28]. Earlier studies have reported excessive iodine intake among 13 countries globally, and the State of Qatar is one of them [29]. Apart from Qatar, the GCC region's remaining countries have reported adequate Iodine status [30].

The literature shows that the people living in coastal areas or consuming more aquatic products had higher UIC, which is consistent with our study findings [18]. In Korea and Japan, iodized salt and iodine‐rich foods like seaweed and fish are popular and commonly consumed in their traditional diet [22, 23]. Our study cohort also reported substantial amounts of seafood consumption. A few participants even reported consuming a seafood source a day before urine sample collection, which might have influenced the UIC levels. Although most individuals tolerate a high dietary iodine intake, studies have shown that prolonged intake can be harmful as it may alter thyroid function [31].

In the present study, most school‐aged children had high UIC levels; however, 6.4% of the subjects showed UIC < 100 μg/L, indicating iodine deficiency. Choi et al. and Lee et al. reported a similar tendency of low iodine in 2.1% and 3.9% of Korean school‐aged children, respectively [22, 26]. Proper evaluation should be done for children with low iodine levels. Literature thus suggests regular UIC screening to prevent iodine deficiency status [18, 19].

The prevalence of goiter is a major indicator of IDD surveillance in school‐aged children [8, 19]. Moreover, it is well documented that the prevalence of goiter in school children is higher in iodine‐excess or ‐deficient areas than in iodine‐sufficient areas [8, 29, 30, 32]. Based on the physical examination of the thyroid gland in the current study, goiter (palpable/visible) was found only in 0.4% of study population. Studies conducted in Ethiopia reported a much higher percentage (16%, 37%, and 62%) of palpable goiter compared to our study [27, 33, 34]. The main reason for this discrepancy could be attributed to the agroecology variation, as the Ethiopian study sample was mainly taken from the highlands [27]. Adequate knowledge about goiter and deficiency disorders and adequate iodine salt use by our study participants were in accordance with the results of Korean and Cambodian studies [11, 24].

### Limitations

One limitation of our study is that it included only 6–12‐year‐old school children, leaving behind the other vulnerable age groups. In the present study, UIC was based on a single random spot urine collection. Although 24‐h urine sample collection is recommended, it is difficult to collect it from all study participants. Nowadays, several spot urine collections with mean UIC values are considered more reliable because of day‐to‐day variation in iodine intake [8, 16]. Blood samples for the thyroid profile were not taken, which could have been correlated with increased UIC. Ultrasound for thyroid size was not performed, which is recommended in some studies. Another limitation can be the recall bias for daily dietary intake. Despite these limitations, the use of urinary iodine levels, goiter grading, and nationwide coverage are the important strengths of this study, which may compensate for these weaknesses. Furthermore, we used a larger sample size with random repeat spot urine samples from the existing cohort, which increases the power of the study.

## CONCLUSION

It is the first report that examined the iodine status in school‐aged children in the State of Qatar. The median UIC results indicated that iodine intake amongst school‐going children exceeds the recommended levels. Hence, multi‐sectoral national efforts are required to bring iodine intake within the recommended cutoff values for the age group under study. This could be attained by reducing the daily salt intake, revising the salt iodine content specification, and providing regular dietary counseling services. The WHA recommends that the iodine status should be monitored every 3 years; within this strategy lies the urgent need to continuously monitor iodine content and salt intake at the population level in the State of Qatar. A systematic approach is required to monitor the salt and iodine intake in the State of Qatar. This will include establishing systems and processes for conducting periodic surveys and regular reporting of salt and iodine consumptions to guide policy decisions.

At an interim level, a repeat survey may be conducted to determine the current salt iodine intake levels in the country and guide immediate actions to prevent overconsumption of iodine. Further research would be necessary to understand the health impacts of excessive iodine intake among population in the State of Qatar.

## AUTHOR CONTRIBUTIONS

*Conceptualization; supervision*: Mohamed Hamad J. T. Al‐Thani. *Conceptualization; supervision; methodology*: Salah Abdulla Sh. A. Alyafei, Kholoud Ateeq K. M. Al‐Motawaa. *Data curation; data analysis; and validation*: Shamseldin Ali Khalifa, Benjamin Vinodson. *Data analysis; writing—original draft, review, and editing*: Syed Hassan Bin Usman Shah. *Writing—original draft; review; and editing*: Sureshbabu Kokku, Amit Mishra. All authors approved the final version submitted.

## CONFLICT OF INTEREST

The authors declare no conflict of interest.

## ETHICS STATEMENT

Ethical clearances for this study were obtained from MoPH and the MEHE. Permission from each school administration was also taken to undertake the survey in their school premises. Informed consent from the parents/guardians was taken as the target population included 6–12‐year‐old primary school‐going children.

## Data Availability

The data that support the findings of this study are available from the corresponding author upon reasonable request.
